# The Influence of Gender and Age on the Outcomes of and Adherence to a Digital Interdisciplinary Mental Health Promotion Intervention in an Australasian Nonclinical Setting: Cohort Study

**DOI:** 10.2196/29866

**Published:** 2021-11-11

**Authors:** Geraldine Przybylko, Darren Morton, Jason Morton, Melanie Renfrew

**Affiliations:** 1 Lifestyle Medicine and Health Research Centre Avondale University Cooranbong Australia; 2 Faculty of Education, Business and Science Avondale University Cooranbong Australia

**Keywords:** age, gender, adherence, digital health, interdisciplinary, mental health, promotion, intervention, lifestyle medicine, positive psychology, multicomponent, lifestyle, outcome, cohort study

## Abstract

**Background:**

The global prevalence of mental health disorders is at a crisis point, particularly in the wake of COVID-19, prompting calls for the development of digital interdisciplinary mental health promotion interventions (MHPIs) for nonclinical cohorts. However, the influence of gender and age on the outcomes of and adherence to MHPIs is not well understood.

**Objective:**

The aim of this study was to determine the influence of gender and age on the outcomes of and adherence to a 10-week digital interdisciplinary MHPI that integrates strategies from positive psychology and lifestyle medicine and utilizes persuasive systems design (PSD) principles in a nonclinical setting.

**Methods:**

This study involved 488 participants who completed the digital interdisciplinary MHPI. Participants completed a pre and postintervention questionnaire that used: (1) the “mental health” and “vitality” subscales from the Short Form 36 (SF-36) Health Survey; (2) the Depression, Anxiety and Stress Scale (DASS-21); and (3) Satisfaction With Life Scale (SWL). Adherence to the digital interdisciplinary MHPI was measured by the number of educational videos the participants viewed and the extent to which they engaged in experiential challenge activities offered as part of the program.

**Results:**

On average, the participants (N=488; mean age 47.1 years, SD 14.1; 77.5% women) demonstrated statistically significant improvements in all mental health and well-being outcome measures, and a significant gender and age interaction was observed. Women tended to experience greater improvements than men in the mental health and well-being measures, and older men experienced greater improvements than younger men in the mental health and vitality subscales. Multiple analysis of variance results of the adherence measures indicated a significant difference for age but not gender. No statistically significant interaction between gender and age was observed for adherence measures.

**Conclusions:**

Digital interdisciplinary MHPIs that utilize PSD principles can improve the mental health and well-being of nonclinical cohorts, regardless of gender or age. Hence, there may be a benefit in utilizing PSD principles to develop universal MHPIs such as that employed in this study, which can be used across gender and age groups. Future research should examine which PSD principles optimize universal digital interdisciplinary MHPIs.

**Trial Registration:**

Australian New Zealand Clinical Trials Registry ACTRN12619000993190; http://www.anzctr.org.au/Trial/Registration/TrialReview.aspx?id=377889 and Australian New Zealand Clinical Trials Registry ACTRN12619001009101; http://www.anzctr.org.au/ACTRN12619001009101.aspx

## Introduction

### Background

The global prevalence of depression and anxiety prior to the COVID-19 pandemic was estimated at 586 million people [1]. In Australia, 1 in 5 individuals reported a mental or behavioral condition in 2017-2018 [2], with the prevalence being higher among women (22%) than men (18%) and in the 15-24 years age range (26%). Recent population-based surveys indicate that mental health has further deteriorated since COVID-19 [3,4]. In response, there is a need for accessible and scalable mental health and well-being interventions that not only support individuals suffering from mental distress but that also promote the mental health and psychological resilience of nonclinical cohorts [5-7].

Emerging literature has shown that digital mental health promotion interventions (MHPIs) can improve mental health [8,9] and prevent the onset of mental health disorders [10,11]. Research suggests several advantages of digital MHPIs, including the potential to target individuals before they reach a diagnostic threshold [12], being more economical than face-to-face interventions [13], increased acceptability, and having the potential to disseminate on a wider scale [10]. The use of mobile apps and texting for self-guided care may help to improve physical health while reducing anxiety, stress, and depression [14]. Conversely, the literature also identifies several disadvantages of digital MHPIs, including the resources spent on individuals who do not develop adverse outcomes [13], higher dropout rates [15], and the smaller effect sizes compared with those of clinical interventions [10]. Digital interventions targeting clinical cohorts have been found to be beneficial for the treatment of acute depression, and a meta-analysis concluded that gender and age are not moderating factors of the outcomes [16]. Digital MHPIs have also been shown to be effective tools for enhancing the mental health and well-being of nonclinical cohorts, although little is known about the influence of gender and age on the responsiveness to these interventions in this context [17].

### Influence of Gender on MHPIs

There is a paucity of research examining the influence of gender on the outcomes of and adherence to digital interdisciplinary MHPIs when delivered in nonclinical settings. It is well established that men and women experience mental health issues and distress differently. For example, women are more likely to talk to someone, seek professional health care, protect themselves and their offspring, and continue engaging with their social networks [18]. In contrast, men typically build up their emotions over time, which may develop into adverse behaviors such as anger, violence, and hostility that in turn can compound the mental health issue [19].

Studies also show that men are typically more hesitant than women to seek help or treatment for mental health concerns [20]. Notably, women tend to rate MHPIs as more important than men [21] and self-selected mental health interventions typically have a bias toward female participation [15,22-27]. Numerous strategies such as role models, incorporating problem-solving tasks, and portraying positive male traits have been proposed for improving the outcomes of MHPIs when targeting men [28]. The use of MHPIs in male-dominated industries has been reported to improve mental health literacy and knowledge, increase social support, provide access to treatment, and reduce absenteeism [29].

Further, men may require more encouragement than women to engage in, and adhere to, digital interventions, thus requiring better implementation of programs [30]. Promoting enjoyable activities and creating sustainable cultures that facilitate group comradery are also deemed positive strategies for MHPIs [31] as they encourage trust, reduce stigma, and normalize engagement [32]. Interestingly, an Australian Football League themed app using young male role models, psychoeducation, social connection, and applied games to target men reported 60-day improvements in flourishing and a sense of connection to the intervention community regardless of gender [33]. However, there is limited understanding of the influence of gender on the outcomes of and adherence to digital interdisciplinary MHPIs, which was the main objective of this study.

### Influence of Age on MHPIs

The literature is also sparse regarding the influence of age on the outcomes of and adherence to digital interdisciplinary MHPIs in a nonclinical setting. Although young adults are commonly termed “digital natives” [34,35], this does not necessarily equate to interest and engagement with digital MHPIs and services. For example, a large web-based survey on university students revealed that those with psychological distress (26.14%, 1577/6034) reported a low utilization rate of 2.98% (47/1577) for online mental health services, despite 59.99% (946/1577) reporting a willingness to use the services [36]. Regardless, digital mental health interventions have been found to be effective for improving depression, anxiety, and psychological well-being among college students. However, further investigations are required to understand the key factors to optimize such interventions [37].

Little is known on the efficacy of digital MHPIs for older adults in a nonclinical setting. New technologies are promising tools to alleviate loneliness and social isolation [38]. Novel interventions such as virtual reality interventions have been found to improve psychological well-being in older adults in a nonclinical setting, and have the potential to foster environmental mastery, personal growth, and autonomy [39]. In addition, animatronic pets provide a promising opportunity to support healthy older adults in reducing loneliness, increasing quality of life, and improving psychological well-being [40].

A key challenge is the ability to develop user-driven, action-based mental health interventions for younger men that shift behavior, stigma, and leverage the influence of their peers [41]. Social influences are more prominent in the adoption of mobile health services among young to middle-aged adults compared with older adults [42].

A key factor affecting adherence to digital mental health interventions among older adults is whether their expectations of the potential outcomes are realistic [43]. Managing expectations reduces the likelihood of older adults deeming the intervention to be a “waste of time” and hence increases adherence, which contributes to improved outcomes of the intervention. Interestingly, mental health interventions are rarely designed with the novice digital user in mind or in accordance with the digital guidelines of older participants [44]. In fact, an “innovativeness-needs paradox” seems to exist where the people in most need of the digital MHPI are those at the highest risk of having the least access, training, skills, adoption rates, and adherence to the intervention, therefore increasing health care inequalities [45]. Digital MHPIs have the potential to reduce the gap in health care provision for older adults as many patients have long wait times for mental health providers, need help in the prevention and management of a multitude of chronic diseases, and have limited access to mental health providers as they are unable to travel long distances [45].

### Persuasive Systems Design

It is well recognized that adherence is problematic in digital MHPIs. Two decades ago, Fogg [46] coined the term “interactive technology” as the design to leverage social influence and motivate and persuade humans to change their attitudes and behaviors. Interactive technology involves features that reward people with positive feedback, model a target behavior or attitude, and provide social support. Oinas-Kukkonen and Harjumaa [47] progressed the work of Fogg by proposing persuasive systems that incorporate information software or systems that are devised to reinforce, change, or formulate attitudes and/or behaviors. In addition, the persuasive systems design (PSD) model was developed specifically to optimize engagement with digital interventions [47]. The PSD model incorporates four categories of persuasion principles: Primary Task Support, Dialogue Support, System Credibility Support, and Social Support. Each category includes 7 distinct persuasion principles, including reduction, tunneling, tailoring, self-monitoring, rewards, reminders, liking, trustworthiness, expertise, surface credibility, social learning, social comparison, and normative influence and completion. Studies have also demonstrated that PSD principles can improve the outcomes of and adherence to digitally delivered interventions [48-52].

Game-based digital mental health interventions were particularly found to increase the participants’ engagement and adherence over the long term [53]. Although gaming is typically used by the younger male demographics, gender and age were not associated with frequency of play [53]. In contrast, a systematic review reported no evidence that the use of gamification was associated with increased adherence to the protocol of the program [54]. However, this may be due to most studies utilizing only one feature of gamification (eg, goal-setting, progress, feedback reward, or story/theme).

Common reasons reported for nonadherence include lack of time, disinterest in the intervention, treatment no longer needed, hardware or technical issues, perceived ineffectiveness of the intervention, life events, chose not to proceed as participants felt better after undertaking a few modules, discontent with the group assignment, holiday, work commitments, poor health, and no longer wished to participate [52].

### Study Objectives

Mental health promotion is crucial for improving population-level mental health. Despite the emerging literature supporting the effectiveness of digital MHPIs [23,25], there is a paucity of research investigating the influence of gender and age in nonclinical cohorts. In this study, we aimed to investigate the influence of gender and age on the outcomes of and adherence to a digital interdisciplinary MHPI in a nonclinical cohort.

## Methods

### Study Design

We previously reported that a randomized controlled trial (RCT) using an MHPI, referred to as the “Live More Project” or “The Lift Project,” showed significant improvements (*P*<.001) in all outcome measures for an intervention group, whereas no changes were observed in the control group regardless of gender and age [25]. The focus of this study was to examine the influence of gender and age on the outcomes of and adherence to a digital MHPI among a larger cohort.

### Study Participants

This study combined the data of two cohorts from two independent studies that utilized the same intervention in an Australasian nonclinical setting (see Table 1), creating a total study population of 488 participants. The participants were recruited voluntarily through a faith-based organization. The study was advertised as an “emotional wellness” program through the faith-based organization’s internal communications channels, including bulletins and magazines.

Cohort 1 was the treatment arm (n=168) from an unblinded RCT. The Avondale University Human Research Ethics Committee approved all procedures involving human subjects for the RCT (project number 2017:13). The trial protocol was registered at the Australian New Zealand Clinical Trials Registry (ACTRN12619000993190). For more information about the study design and intervention, refer to Przybylko et al [25].

**Table 1:** Overview of weekly topics and challenges for the intervention.

Week	Topic	Daily challenge	Weekly challenge
1	Speak positively	Offer a genuine compliment	Memorize an inspirational text or saying
2	Move dynamically	Spend 30 minutes of moderate exercise or 10,000 steps	20 minutes of guided resistance exercises
3	Immerse in an uplifting natural environment	Spend 30 minutes in an uplifting natural environment	Experience a sunrise
4	Immerse in a positive social environment	Do something intentional to show you care	Forgive someone who has hurt you
5	Look to the positive	Spend 15 minutes to reflect on three things that went well	Write a letter of gratitude to someone and share it with them
6	Eat nutritiously	Eat eight servings of plant-based food	Prepare a high-fiber, plant-based meal with one or more friends
7	Rest: sleep	Spend 8 hours in bed without a device	Spend an evening by firelight
8	Rest from stress	Spend 15 minutes in a quiet place, relaxing, and being mindful of surroundings	Take a day off work and a digital Sabbath (going “offline” for 24 hours to recharge)
9	Serving others	Perform a random act of kindness	Use signature strength to perform an act of service
10	What does it take to flourish?	Continue challenges found to be helpful	Continue challenges found to be helpful

Cohort 2 involved a three-arm (n=320) randomized comparative study that examined the influence of different modes of human support on the intervention. The Avondale University Human Research Ethics Committee approved the conduct of the study (project number 2018:09) and the trial was registered at the Australian New Zealand Clinical Trials Registry (ACTRN12619001009101); refer to Renfrew et al [23] for a detailed explanation of the study. The study indicated that the intervention improved the mean scores for all mental health metrics, regardless of the addition of human support. Further, the mode of human support offered in addition to the intervention had no influence on the outcomes of the intervention [55]. Hence, as the mental health outcomes were similar for all arms in the study, all participants were pooled to form Cohort 2.

### Intervention

Both cohorts participated in an intervention referred to as the “The Live More Project” or “The Lift Project” [56,57]. The 10-week digital interdisciplinary MHPI used evidenced-based strategies from the disciplines of lifestyle medicine and positive psychology for improving mental health and well-being, as detailed in Table 1.

The intervention was based on Ajzen’s [58] Theory of Planned Behavior and employed an experiential pedagogical framework of “Learn, Experience, Think, Share,” which was accessed through an electronic learning management system. Weekly 30-minute video sessions were aimed to educate and empower participants to make positive behavior changes. Daily and weekly experiential challenges provided practical application. The following PSD principles were used in the intervention to improve adherence: gamification to increase challenge points and badges by participating in the challenges; a social forum to comment, post photos, and encourage interaction between the participants to provide accountability; and provision of reminders to watch the videos and log challenges. Refer to Multimedia Appendix 1 for website and app screenshots. An electronic book and electronic workbook were also provided to expand the participants’ knowledge and to journal their experience during the intervention.

### Measurements

#### Mental Health Outcomes

All participants in each cohort completed a self-reported questionnaire at preintervention (Week 1) and postintervention (Week 12). The questionnaire included sociodemographic characteristics such as age, gender, ethnicity, level of education, country of birth, and three validated instruments. An outline of the instruments used in the questionnaire are detailed below.

#### Short Form 36-Item Health Survey

The Short Form 36-item (SF-36) Health Survey is a self-reported health questionnaire appropriate for use among a general population [59]. This survey consists of 36 items that assess eight scales: general health, mental health, vitality, social function, physical function, role limitations due to physical health, role limitations due to emotional problems, and bodily pain. These subscales can be used separately; the two subscales measuring positive affect (ie, “mental health” and “vitality”) [60] were used in this study. The “mental health” subscale assesses emotional well-being (5 items) and the “vitality” subscale assesses energy and fatigue (4 items) [61]. Both subscales generate a score between 0 and 100, with a higher score representing a higher level of mental health and vitality. Although exact cut-off scores have not been established for the two subscales, studies have indicated that a mental health score less than 56 is indicative of major depression [62] and a score less than 45 on the vitality subscale was classified as fatigued [63]. This study observed a Cronbach α of .86 for mental health and .88 for vitality, indicating good internal consistency.

#### Depression, Anxiety and Stress Scale

The 21-item Depression, Anxiety and Stress Scale (DASS-21) is a well-used assessment to measure the negative affect of emotional states—depression, anxiety, and stress (7 items per subscale)—on both clinical and nonclinical populations [64]. The questionnaire generates a score between 0 and 21, with a higher score representing increasing levels of mental distress [65]. Individuals were considered “symptomatic” if they reached the threshold of greater than 4 for depression, greater than 3 for anxiety, and greater than 7 for stress. This study observed good internal consistency with a Cronbach α of .87 for depression, .70 for anxiety, .83 for stress, and .90 for the overall score for the 3 domains.

#### Satisfaction With Life Scale

The 5-item Satisfaction With Life Scale (SWLS) assesses global life satisfaction [66] and is used in numerous settings [67]. The questionnaire generates a score between 5 and 35, with a higher score representing increasing levels of life satisfaction. A score of 19 indicates an average life satisfaction. This study observed a Cronbach α of .88, indicating good internal consistency.

### Measurements

#### Adherence

This study used the following adherence measures for the intervention that have been established previously [55].

#### Videos Viewed

Each week the participants were introduced to a weekly topic (see Table 1) that was presented using an educational video. The total number of weekly videos viewed was used to measure primary adherence and was measured out of a total of 10. A video was marked as “viewed” when 80% or more of the presentation had been played.

#### Experiential Challenge Activities

Participants were encouraged to put what they had learned each week into action by participating in experiential challenge activities. Adherence to challenges was calculated through the total weekly challenge score and the total number of weeks that the participants had completed the challenge. The daily challenge was awarded 10 points with a maximum of 70 points per week, and weekly challenges were allocated 30 points. Hence, participants had the opportunity to achieve 100 points per week, for a total of 1000 points at the end of the 10-week intervention.

### Statistical Analysis

The data were analyzed using SPSS Statistics (version 25). The *χ^2^* test was used to examine the difference in the baseline characteristics. Descriptive statistics, involving frequencies, means, SDs, and 95% CIs, are used to present the mental health and well-being outcomes, as well as the adherence measures.

Multivariate analysis of variance (MANOVA) was used for comparisons as there were several categorical independent variables and continuous dependent variables. Data were prescreened and cleaned to ensure the robustness of the MANOVA, which was also aided by the large sample size. MANOVA, using the general linear modeling (GLM) function in SPSS, was used to test for time effects (pre to postintervention), gender and age effects, and their interactions. When significant, Bonferroni post hoc analyses were utilized to determine significant changes from pre to postintervention to compare gender and age differences, and to explore significant interactions. Pearson correlation analysis was used to evaluate the relationship between the mental health or well-being outcomes and adherence measures. Analysis of variance was used to compare differences in the outcomes and adherence measures between the age categories. Paired and independent sample *t* tests were used to explore gender differences in the mental health and well-being outcome measures. Missing data for age (n=4) were replaced with the mean age and missing data (n=14) for mental health outcomes were removed from the analysis.

## Results

### Participant Baseline Characteristics

A total of 488 participants completed the preintervention questionnaire (week 1) and postintervention questionnaire (week 12).

Cohorts 1 and 2 differed with regard to age (mean 49.3 years, SD 14.1 and 45.9 years, SD 14.0, respectively*; P*=.01), gender balance (women: 69.1%, 116/168 and 81.9%, 262/320, respectively; *P*=.002), and ethnic representation (White: 89.3%, 150/168 and 81.8%, 262/320, respectively; *P*=.05). Although a statistically significant difference was observed between the cohorts in these demographic variables, it is notable that in both cohorts there was a bias toward White women in the 35-54–year age category. No statistically significant difference was found between Cohorts 1 and 2 in the highest education obtained (tertiary education: 89.3% and 81.8%, respectively; *P*=.52).

There was a difference between Cohorts 1 and 2 in all baseline mental health measures except life satisfaction; however, the mean scores for both cohorts were in the nonclinical range for all measures. The baseline mental health measures for the two cohorts were as follows: mental health (75.5 and 66.2, *P*<.001), vitality (52.5 and 60.2, *P*<.001), depression (2.5 and 3.5, *P*=.001), anxiety (1.8 and 2.3, *P*=.02), stress (4.5 and 5.7, *P*=.001), and life satisfaction (23.9 and 23.1, *P*=.08). Combining these two cohorts increased the heterogeneity of the total sample, which in turn increased the generalizability of the study.

The combined cohort (N=488), which formed the population for this study, had a mean age of 47.1 years (SD 14.1) and were mostly women (77.9%, 380/488). The ethnicity of the population was largely White (83.4%, 407/488), followed by Other (5.3%, 26/488), Asian (4.5%, 22/488), Maori/Pacific Islander (3.3%, 16/488), Black/African American (2.3%, 11/488), Spanish/Hispanic/Latino (0.6%, 3/488), and Indigenous (0.6%, 3/488). The highest level of education achieved was tertiary education (84.4%, 413/488), followed by secondary/high school (15.2%, 75/488) and primary/elementary (0.4%, 2/488).

In the absence of standardized or universally accepted age categorization, the authors determined three age categories based on the age grouping system of the World Health Organization [68]: 18-34 years (younger adults: 21.7%, 106/488), 34-54 years (middle-aged adults: 47.1%, 230/488), and ≥55 years (older adults: 31.1%, 152/488). However, the World Health Organization acknowledges that there is no conceptual justification for selecting one age standard over another [68].

There was no statistically significant difference between men and women (*χ^2^*_487_=1.42, *P*=.70) or the age categories (*F*_487_=0.30, *P*=.69) with regard to ethnic representation (*F*_487_=0.03, *P*=.98) or highest level of education obtained (*F*_487_=0.23, *P*=.59). However, statistically significant differences were found between genders and between age categories in some of the preintervention psychometric measures (see Table 2). At preintervention, women reported poorer mental health metrics than men for mental health (t_487_=4.85, *P*<.001), vitality (t_487_=3.94, *P*<.001), depression (t_487_=–3.13, *P*=.002), anxiety (t_487_=–3.05, *P*=.002), and stress (t_487_=–4.14, *P*<.001), but not life satisfaction (t_487_=1.09, *P*=.29). However, the mean scores were found to be in the nonclinical range. The ≥55 years age category had a significantly better score than the 18-34 (*P*<.001) and 34-54 (*P*=.002) age categories for mental health; the 18-34 (*P*<.001) and 34-54 (*P*<.001) categories for vitality; the 18-34 category for depression (*P*<.001) and anxiety (*P*=.002); and the 18-34 (*P*<.001) and 34-54 (*P*<.001) categories for stress. The 35-54 age category had a significantly higher score than the 18-34 age category for depression (*P*=.03) and anxiety (*P*=.03), but not for stress (*P*=.97).

**Table 2:** Pre to postintervention changes in each of the outcome measures defined by the gender and age categories.

Outcome measure				Preintervention (week 1), mean (SD)		Postintervention (week 12), mean (SD)		Difference, mean (%)		*t* test (*df*)		95% CI		*P* value		Effect size (*Cohen d*)
**Mental health**																
	Overall			69.4 (16.4)		77.9 (14.9)		8.5 (12)		–13.306 (487)		–9.72 to –7.22		<.001		0.54
	**Gender**															
		Men	76.0 (14.4)		81.3 (16.2)		5.3 (7)		–3.227 (107)		–8.59 to –2.05		.002		0.35	
		Women	67.5^a^ (16.5)		76.9 (14.4)		9.4 (14)^a^		–14.197 (379)		–10.69 to –8.09		<.001		0.61	
	**Age category (years)**															
		18-34	64.1 (16.6)		71.3 (17.8)		7.2 (11)		–1.304 (105)		–10.66 to –3.83		<.001		0.42	
		35-54	68.5 (15.3)		77.0 (14.1)		8.4 (12)^b^		–9.695 (229)		–10.13 to –6.71		<.001		0.57	
		55+	74.3^b,c^ (16.7)		83.7 (11.7)		9.5 (13)^b^		–9.182 (151)		–11.50 to –7.43		<.001		0.67	
**Vitality**																
	Overall			57.6 (18.2)		86.2 (17.6)		10.5 (18)		–14.018 (487)		–12.01 to –9.06		<.001		0.59
	**Gender**															
		Men	63.5 (16.7)		69.9 (19.9)		6.4 (10)		–3.416 (107)		–10.06 to –2.67		<.001		0.35	
		Women	55.9^a^ (18.3)		67.7 (16.8)		11.8 (21)^a^		–14.772 (379)		–13.31 to –10.19		<.001		0.67	
	**Age category (years)**															
		18-34	53.9 (15.7)		63.0 (19.0)		9.1 (17)		–4.737 (105)		–12.96 to –5.31		<.001		0.53	
		35-54	55.6 (18.7)		66.6 (17.3)		11.1 (20)^b^		–10.647 (229)		–13.14 to –9.04		<.001		0.62	
		55+	63.2^b,c^ (17.8)		73.9 (15.4)		10.7 (17)		–8.475 (151)		–13.21 to –8.21		<.001		0.64	
**Depression**																
	Overall			3.2 (3.3)		1.9 (2.6)		–1.3 (–39)		10.575 (487)		1.02 to 1.49		<.001		0.43
	**Gender**															
		Men	2.3 (3.0)		1.5 (2.7)		–0.8 (–34)		3.464 (107)		0.34 to 1.26		.001		0.28	
		Women	3.4^a^ (3.3)		2.0 (2.6)		–1.4 (–40)^a^		10.121 (379)		1.12 to 1.66		<.001		0.47	
	**Age category (years)**															
		18-34	4.1 (3.5)		2.6 (3.3)		–1.6 (–38)		5.675 (105)		1.03 to 2.14		<.001		0.47	
		35-54	3.1 (3.1)		2.0 (2.6)		–1.1 (–35)		6.562 (229)		0.76 to 1.41		<.001		0.38	
		55+	2.7^b^ (3.2)		1.4 (1.9)		–1.3 (–49)		6.095 (151)		0.88 to 1.72		<.001		0.50	
**Anxiety**																
	Overall			2.1 (2.3)		1.3 (1.8)		–0.8 (–38)		9.242 (487)		0.64 to 0.98		<.001		0.39
	**Gender**															
		Men	1.5 (1.9)		0.9 (1.7)		–0.6 (–42)		3.896 (107)		0.32 to 0.97		<.001		0.36	
		Women	2.3^a^ (2.4)		1.5 (1.8)		–0.9 (–35)		8.382 (379)		0.65 to 1.05		<.001		0.40	
	**Age category (years)**															
		18-34	2.8 (2.7)		1.6 (2.1)		–1.2 (–42)		4.886 (105)		0.70 to 1.67		<.001		0.49	
		35-54	2.1^b^ (2.2)		1.3 (1.9)		–0.7 (–35)		6.574 (229)		0.51 to 0.94		<.001		0.35	
		55+	1.8^b^ (2.1)		1.1 (1.4)		–0.7 (–39)		4.516 (151)		0.38 to 0.98		<.001		0.39	
**Stress**																
	Overall			5.3 (3.4)		3.8 (3.1)		–1.5 (–28)		11.313 (487)		1.24 to 1.76		<.001		0.47
	**Gender**															
		Men	4.2 (2.8)		3.0 (3.2)		–1.2 (–28)		4.270 (107)		0.63 to 1.73		<.001		0.39	
		Women	5.7^a^ (3.4)		4.1 (3.0)		–1.6 (–28)		10.554 (379)		1.30 to 1.89		<.001		0.50	
	**Age category (years)**															
		18-34	6.4 (3.6)		4.9 (3.8)		–1.5 (–24)		4.812 (105)		0.90 to 2.17		<.001		0.42	
		35-54	5.5 (3.1)		4.1 (2.9)		–1.4 (–26)		7.909 (229)		1.08 to 1.79		<.001		0.48	
		55+	4.3^b,c^ (3.4)		2.7 (2.3)		–1.6 (–37)		6.477 (151)		1.09 to 2.04		<.001		0.55	
**Life satisfaction**																
	Overall			23.4 (6.8)		25.8 (6.4)		2.4 (10)		–11.991 (487)		–2.85 to –2.04		<.001		0.37
	**Gender**															
		Men	24.0 (6.5)		26.2 (6.0)		2.2 (9)		–5.694 (107)		–3.02 to –1.46		<.001		0.35	
		Women	23.2 (6.8)		25.7 (6.5)		2.5 (11)		–10.562 (379)		–2.97 to –2.04		<.001		0.38	
	**Age category (years)**															
		18-34	23.3 (7.2)		25.7 (6.7)		2.4 (10)		–5.114 (105)		–3.33 to –1.47		<.001		0.35	
		35-54	22.9 (7.0)		25.7 (6.6)		2.8 (12)		–9.206 (229)		–3.38 to –2.19		<.001		0.41	
		55+	24.0 (6.2)		26.0 (5.9)		2.0 (8)		–5.864 (151)		–2.63 to –1.30		<.001		0.33	

^a^Significant gender difference.

^b^Significant difference from the 18-34 age category.

^c^Significant difference from the 35-54 age category.

### Mental Health Outcomes

#### Overall Intervention Effect

MANOVA results of the changes in the mental health and well-being outcomes from pre to postintervention indicated a statistically significant difference for gender (*F*_487_=2.81, *P*=.01, Wilks Λ=0.97, η^2^=0.03) and age (*F*_487_=2.46, *P*=.004; Wilks Λ=0.94, η^2^=0.03). A significant gender and age interaction (*F*_487_=2.14, *P*=.01; Wilks Λ=0.95, η^2^=0.03) was observed, with younger females experiencing greater improvements than the older females in 5 out of 6 outcome measures. This trend was not evident among the males. Table 2 shows the changes in mental health and well-being outcomes from pre to postintervention, reported for gender and the age group categories. Statistically significant improvements in all mental health and well-being measures were observed.

#### Influence of Gender on Mental Health Outcomes

Although women reported lower levels of mental health (ie, higher emotional distress) at preintervention, they experienced a higher mean change than men in mental health (*F*_487_=13.16, *P*<.001), vitality (*F*_487_=11.90, *P*=.001), and depression (*F*_487_=3.89, *P*=.05), as seen in Table 2. No significant differences were observed between men and women with respect to anxiety (*F*_487_=0.87, *P*=.35), stress (*F*_487_=0.88, *P*=.35), or life satisfaction (*F*_487_=3.53, *P*=.06).

#### Influence of Age on Mental Health Outcomes

Although the ≥55-year age category had higher levels of mental health (ie, lower emotional distress) at preintervention, they experienced a significantly higher mean change in the mental health scale (*F*_487_=5.15, *P*=.006) than the younger age categories. However, there were no statistically significant differences between the age categories for vitality (*F*_487_=2.05, *P*=.13), depression (*F*_487_=0.53, *P*=.58), anxiety (*F*_487_=1.53, *P*=.22), stress (*F*_487_=0.32, *P*=.73), or life satisfaction (*F*_487_=2.15, *P*=.12). The pre to postintervention results indicated that the 18-34–year age category had a significantly lower mean change than the 35-54 (*P*=.009) and ≥55 (*P*=.002) age categories for mental health, and the 35-54 age category for vitality (*P*=.05). Despite the 18-34 age category achieving a higher score (ie, indicating higher emotional distress) at postintervention compared to the 35-54 age category for depression (*P*=.31), anxiety (*P*=.14), and stress (*P*=.54), no statistically significant differences were observed. There were also no statistically significant differences found between the 18-34 and 35-54 age categories for life satisfaction (*P*=.06), or between the 35-54 and ≥55 age categories for any outcome measures: mental health (*P*=.37), vitality (*P*=.80), depression (*P*=.65), anxiety (*P*=.67), stress (*P*=.50), and life satisfaction (*P*=.16). Every age category for both genders showed a statistically significant improvement (ie, lower emotional distress) in mental health and well-being metrics, except for the 18-34–year age category for mental health (*P*=.13) and vitality (*P*=.13), and the men in the ≥55-year age category for stress (*P*=.09).

### Adherence

#### Overall Intervention Effect

MANOVA results of the adherence measures indicated a statistically significant difference for age (*F*_487_=2.20, *P*=.04; Wilks Λ=0.97, η^2^=0.01), but not gender (*F*_487_=1.25, *P*=.29; Wilks Λ=0.99, η^2^=0.01). No statistically significant interaction between gender and age (*F*_487_=0.75, *P*=.61; Wilks Λ=0.99, η^2^=0.01) was observed.

#### Influence of Gender on Adherence

As shown in Table 3, there was no statistically significant gender difference in the number of videos watched (t_487_=–0.52, *P*=.61), total challenge points achieved (t_487_=–1.44, *P*=.15), or the number of weeks that challenges were engaged with (t_487_=–1.72, *P*=.09). Although women recorded higher mean challenge points and a higher percentage watched all 10 videos, there was no significant difference between the genders.

**Table 3:** Adherence outcomes by gender.

Variable			Men (n=108)		Women (n=380)		Total (N=488)		Between-group difference, *P* value
**Number of videos viewed (%)**									N/A^a^
	10	22		33		31			
	8-9	5		3		3			
	5-7	52		39		43			
	1-4	16		20		19			
	0	5		5		5			
Number of videos viewed, mean (SD)			6.4 (2.9)		6.6 (3.2)		6.55 (3.18)		.61
**Challenge, mean (SD)**									
	Challenge points (out of 1000)	355.6 (370.6)		412.2 (361.2)		377.5 (354.0)		.15	
	Number of weeks challenge scores logged (out of 10)	4.4 (3.72)		5.1 (3.63		4.8 (3.6)		.09	

^a^N/A: not applicable.

#### Influence of Age on Adherence

A statistically significant difference was observed between the age categories in the number of videos watched (*F*_487_=5.99, *P*=.003); however, as shown in Table 4, there was no significant difference in the total challenge points (*F*_487_=2.448, *P*=.09) or total number of weeks that challenges were recorded (*F*_487_=2.563, *P*=.08). The age categories of 35-54 and ≥55 years recorded the same mean number of videos watched, which was higher than that for the 18-34 years age category. Both the mean challenge score and the number of weeks that challenge scores were logged showed improvements with age, although the difference was not statistically significant.

**Table 4:** Adherence outcomes between age categories.

Variable			18-34 years (n=106)		35-54 years (n=230)		≥55 years (n=152)		Total (N=488)		Between-group difference, *P* value
**Number of videos viewed (%)**											N/A^a^
	10	25		33		31		89			
	8-9	1		4		3		8			
	5-7	38		42		45		125			
	1-4	26		18		18		62			
	0	10		3		4		17			
Number of videos viewed, mean (SD)			5.6 (3.51)		6.8 (3.06)		6.8 (2.99)		6.6 (3.17)		.01
**Challenge, mean (SD)**											
	Challenge points (out of 1000)	340.8 (346.3)		398.7 (339.0)		442.9 (372.2)		400.3 (363.7)		.09	
	Number of weeks challenge scores logged (out of 10)	4.4 (3.64)		5.0 (3.65)		5.4 (3.62)		5.0 (3.65)		.08	

^a^N/A: not applicable.

## Discussion

### Principal Results

The aim of this study was to investigate the influence of gender and age on the outcomes of and adherence to a digital interdisciplinary MHPI in a nonclinical cohort. To the authors’ knowledge, this study is the first to investigate the effect of gender and age on the outcomes of a digital interdisciplinary MHPI that employed an array of strategies from the disciplines of lifestyle medicine and positive psychology in a nonclinical Australasian setting. Stratification by gender and age showed significant improvements in all mental health and well-being outcomes. Hence, digital interventions such as those employed in this study are useful across gender and age groups for mental health promotion and building psychological resilience.

A female bias was observed in this study, which is consistent with the literature of positive psychology interventions as mentioned previously. Despite the population scoring in the nonclinical range for mental health and well-being, women reported significantly lower mental health scores (ie, higher emotional distress) than men at baseline. However, the women experienced greater improvements than men in the mental health, vitality, depression, and life satisfaction measures. Notably, the women experienced twice the mean change increase in the mental health and vitality subscales compared with the change reported by men, resulting in similar outcome scores to the men at postintervention. This indicates that those scoring lower in the mental health and well-being outcomes can achieve higher mean changes, presumably as there is greater potential for improvement. This is consistent with the results of our previous study using the same intervention that reported higher levels of change were experienced by those with the lowest mental health score at preintervention [57].

This study observed small to medium effect sizes for gender and age on mental health outcomes of a digital interdisciplinary MHPI, which is consistent with the literature of universal digital mental health interventions [10]. However, Tan et al [69] asserts that the impact of small effect sizes can be large when translated to a population level. Hence, digital interdisciplinary MHPIs provide a potential strategy to deliver low-cost and scalable interventions to build the psychological resilience of an individual to help them cope with the adversities experienced in life [70].

This study showed that those aged ≥55 years achieved better mental health and well-being outcomes than the younger age categories in all mental health metrics from pre to postintervention, except for stress. In addition, older men experienced greater improvements than the younger men in the mental health and vitality subscales of the SF-36. These findings are consistent with a meta-analysis of positive psychology interventions, indicating that mental health benefits increased with age [71]. However, these finding are counterintuitive, as younger adults are more frequent users of the internet than older adults (ie, “digital natives”), which could be hypothesized to influence the outcomes of a digitally delivered program [72-74]. A modulating factor might be the time availability. In a previous qualitative study [75], we reported that “time” was perceived as a major barrier to adherence for many participants, although the older participants expressed that retirement provided them with more time to adhere to the intervention. Notably, outcomes of a digital mental health intervention were shown to be related to higher levels of adherence such as higher levels of time spent on the digital platform, number of sessions completed, percentage of the program viewed, and number of activities compared to the control group [76].

Moreover, this study found no significant differences in any adherence measures across gender and age, except for older adults who watched a significantly higher mean number of videos than younger adults. This is consistent with a meta-analysis showing that age was not a predictor of adherence in 13 out of 18 trials [77]. However, this contrasts with a systematic review that found gender to be a consistent predictor of adherence, with women having a higher probability to complete the intervention compared with men [78]. Nevertheless, the authors acknowledged that higher preintervention scores for depression and low scores in anxiety were also found to predict greater adherence.

Naslund et al [79] suggested that focusing digital technologies on early intervention for younger people is key for advancing global mental health. However, Forsman et al [17] argues that the implementation and innovation of mental health promotion for older adults must not be overlooked. Mental health promotion for older adults is of particular importance for three key reasons: there is a higher mental health burden of disease for older adults, digital mental health solutions can improve the mental health care of older adults, and it is well recognized that the mental health of young individuals is strongly influenced by the well-being of their older caregivers [72].

As this study observed improvements in the mental health outcomes of and adherence to a digital interdisciplinary MHPI regardless of gender and age, the authors challenge the concept of focusing solely on mental health promotion for younger or older adults. Instead, the authors encourage developers to be strategic and design digital interdisciplinary MHPIs for all adults (ie, universal). The intervention used in this study employed strategies to increase engagement and adherence among men (ie, using male role models, portraying positive male traits, promoting enjoyable activities, and facilitating peer involvement), younger adults (ie, action-based intervention and leverage the influence of peers), and older adults (ie, designing the intervention for the novice user and managing expectations of the intervention).

In addition, principles from the established PSD categories were incorporated into the intervention. First, from the Primary Task Support category, “reduction,” “tunneling,” and “self-monitoring” were used to aid adoption by novice users and older adults, and to increase adherence by encouraging behavioral change through participation in a variety of challenges (ie, enjoyable activities). Second, from the Dialogue Support category, the PSD principles of “rewards,” “reminders,” and “liking” were incorporated in the MHPI to increase adherence in the form of alerts and personalized reminders [32,49,54]. Third, from the System Credibility Support category, the PSD principles of “trustworthiness,” “expertise,” and “real-world feel” were incorporated by using an internationally recognized male role model that provided credibility to the intervention to build trust and to portray positive male traits. Lastly, from the Social Support category, the PSD principles of “social learning,” “social comparison,” “social facilitation,” and “competition” were employed to promote peer involvement through social interaction (ie, encourage participants to write comments and post pictures in relation to the challenges) and increase adherence and accountability through the use of gamification (ie, points, badges, and the leaderboard). The culmination of the design elements [31,43,80,81] incorporated in the intervention resulted in it being effective; however, further research should investigate which elements are most beneficial and for whom.

### Strengths and Limitations

The strengths of this study are outlined below. First, this study is strengthened by a large number of participants (N=488) and vast age range (18-88 years old) across geographically diverse areas. The second main strength is the MHPI’s novel interdisciplinary nature that utilized multicomponent, evidenced-based strategies from the disciplines of lifestyle medicine and positive psychology. Using multicomponent strategies, rather than employing a single tactic, is also deemed to be more efficacious [56,71,82]. Third, the use of PSD principles in the intervention were both gender-responsive and age-sensitive. Increasing the number of PSD principles does not necessarily lead to better outcomes [52]. Future studies could investigate which PSD principles best optimize universal MHPIs.

There are also several limitations of the study. First, the participants were self-selected and drawn from a faith-based population. Hence, they may have entered the study with higher motivation levels and readiness for change than the general population, which may accordingly limit the generalizability of the findings. Second, there was a female bias to the study—which is often observed in positive psychology interventions—and may limit the generalizability of the intervention to male participants. Future studies could explore the use of male-centric advertising and recruitment locations to increase the number of male participants. Third, the study observed small to medium effect sizes for gender and age, which is consistent with the literature. Lastly, as the intervention was promoted as a mental well-being intervention, the sample was in the “nonclinical range” for the mental health scores. Therefore, further research will need to be undertaken to investigate the influence of gender and age on the outcomes of and adherence to digital mental health interventions that integrate strategies from positive psychology and lifestyle medicine when dealing with clinical populations. 

### Conclusions

The findings of this study demonstrate that a digital interdisciplinary MHPI that employed multicomponent evidence-based strategies from the disciplines of lifestyle medicine and positive psychology using PSD principles can significantly improve mental health and well-being outcome measures across gender and age categories in a nonclinical setting. There may be a benefit in utilizing PSD principles to develop universal MHPIs such as that employed in this study, which can be used across gender and age groups. Future research should examine which PSD principles optimize a universal digital interdisciplinary MHPI.

## Appendix

Multimedia Appendix 1 Website and app screenshots.

## Abbreviations

DASS: Depression, Anxiety and Stress Scales
MANOVA: multivariate analysis of variance
MHPI: mental health promotion intervention
PSD: persuasive system design
RCT: randomized controlled trial
SF-36: Short Form 36 Health Survey
SWLS: Satisfaction With Life Scale
