# Supplementary material for: The Influence of Gender and Age on the Outcomes of and Adherence to a Digital Interdisciplinary Mental Health Promotion Intervention in an Australasian Nonclinical Setting: Cohort Study
Source: JMIR Ment Health. 2021 Nov 11;8(11):e29866. doi: 10.2196/29866 (PMC8663468; doi:10.2196/29866)
Supplement: Multimedia Appendix 1 [file mental_v8i11e29866_app1.pdf]

These images portray features of the website e-learning management system and mobile app relating to the following article:

The Influence of Gender and Age on the Outcomes of and Adherence to an Interdisciplinary Digital Mental Health Promotion Intervention in an Australasian Nonclinical Setting: A Cohort Study

Authors: Przybylko G, Morton J, Morton D, Renfrew M

Section 1:

The Website

Images of the website 2018
(e-learning management system)

https://eliawellness.com/

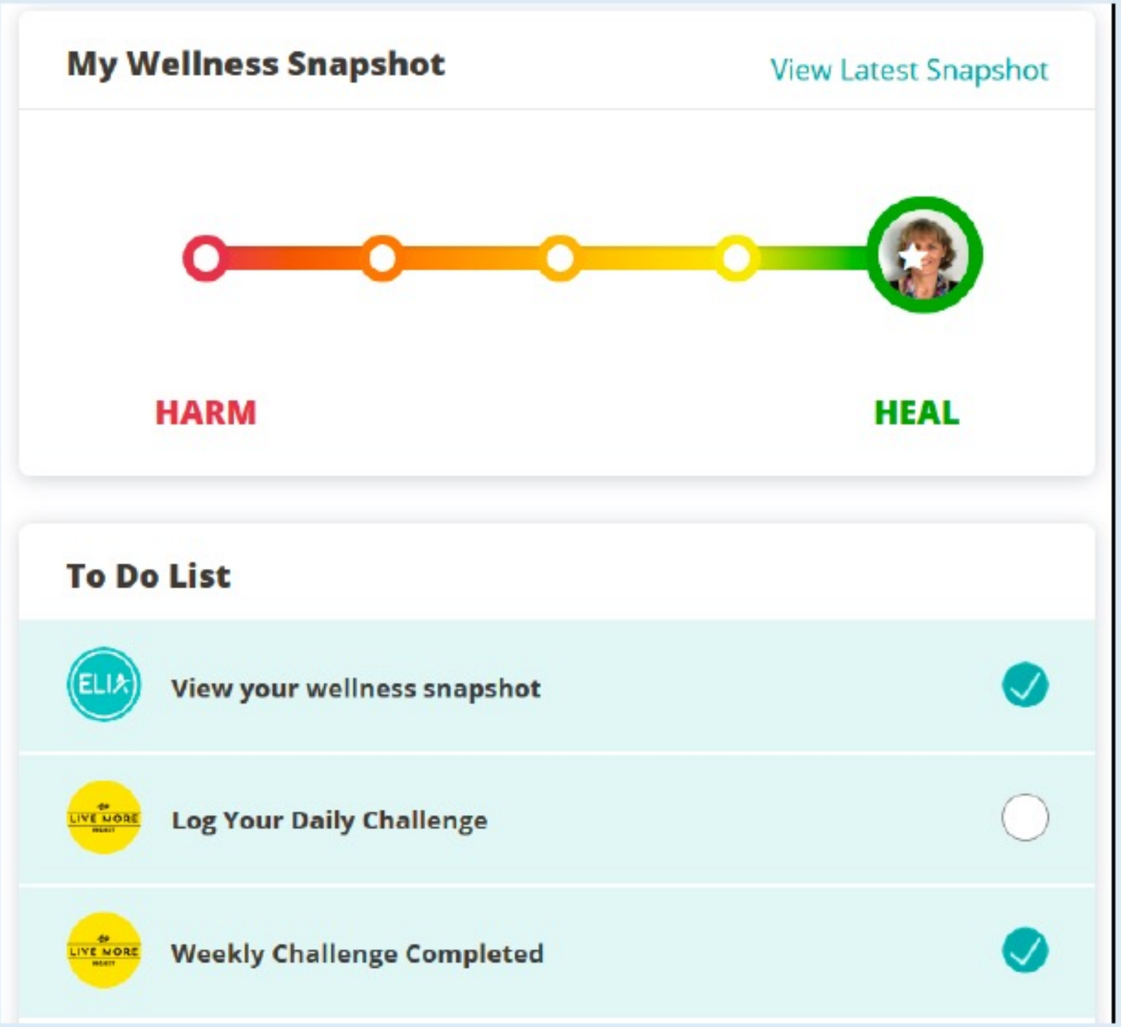

To Do List: An online "To Do" list (available on each personal dashboard) provided easily accessible links to complete the various components. Note: the "wellness snapshot" refers to the prequestionnaire administered at baseline and again on completion (postquestionnaire) of the program.

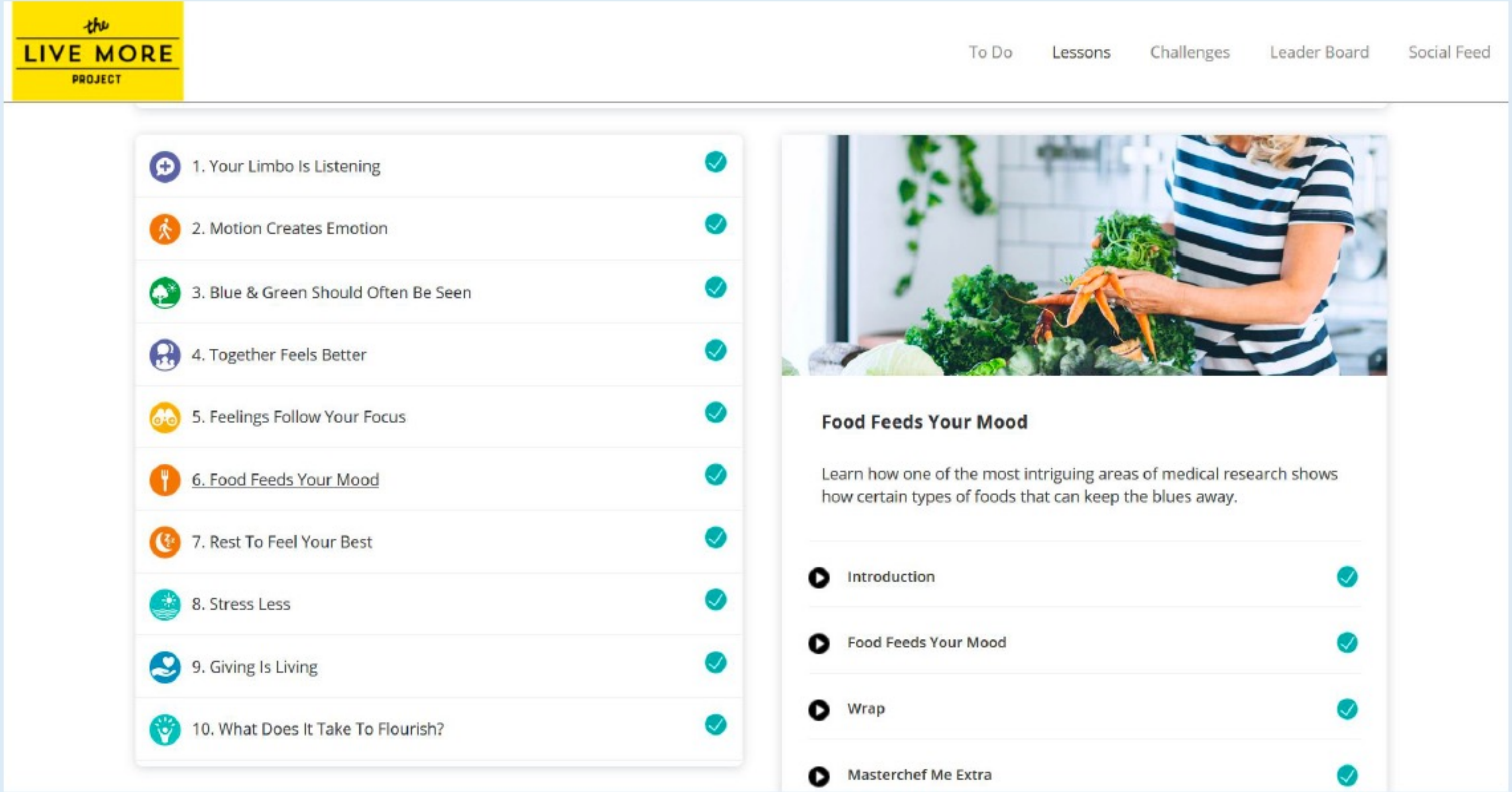

Sample View of Lesson 6 Content: Each lesson was divided into 3-4 short video presentations. Each new video segment was unlocked, on completion of the previous video. Segments were ticked off automatically as they were viewed.

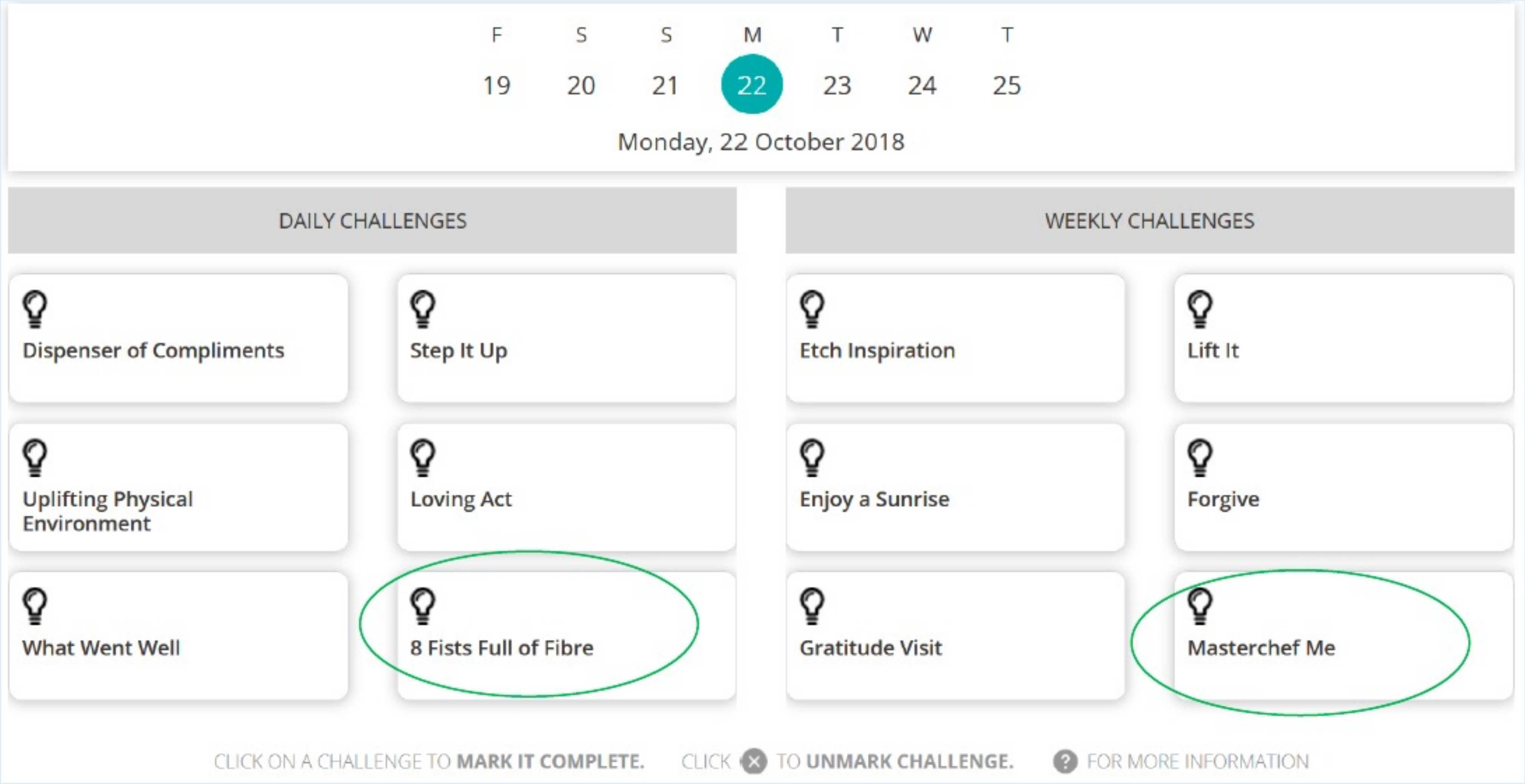

View of challenge icons for lessons 1-6: After each lesson, participants are provided with daily and weekly challenges. For example, for Lesson 6, participants were challenged to eat 8 fists full of fibre daily, and were asked to make 1 high-fibre plant based meal for the weekly challenge - “Masterchef Me”.

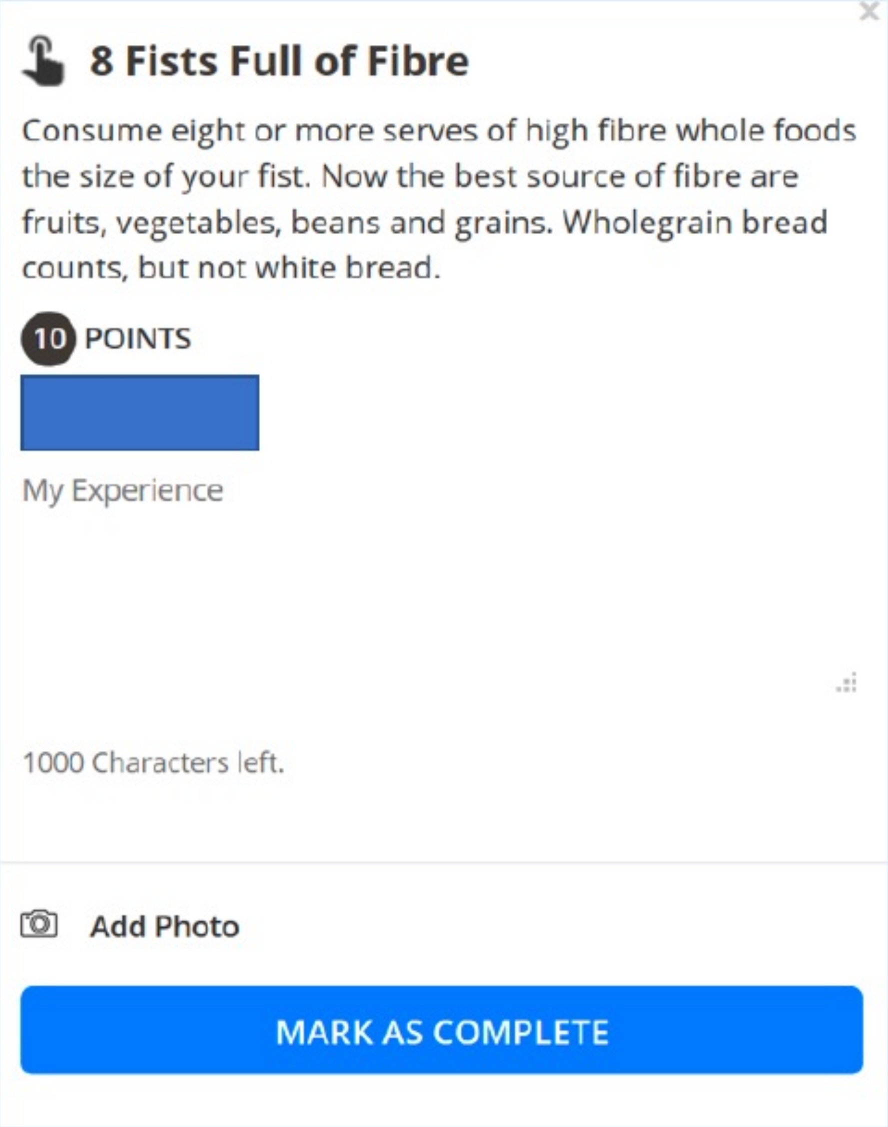

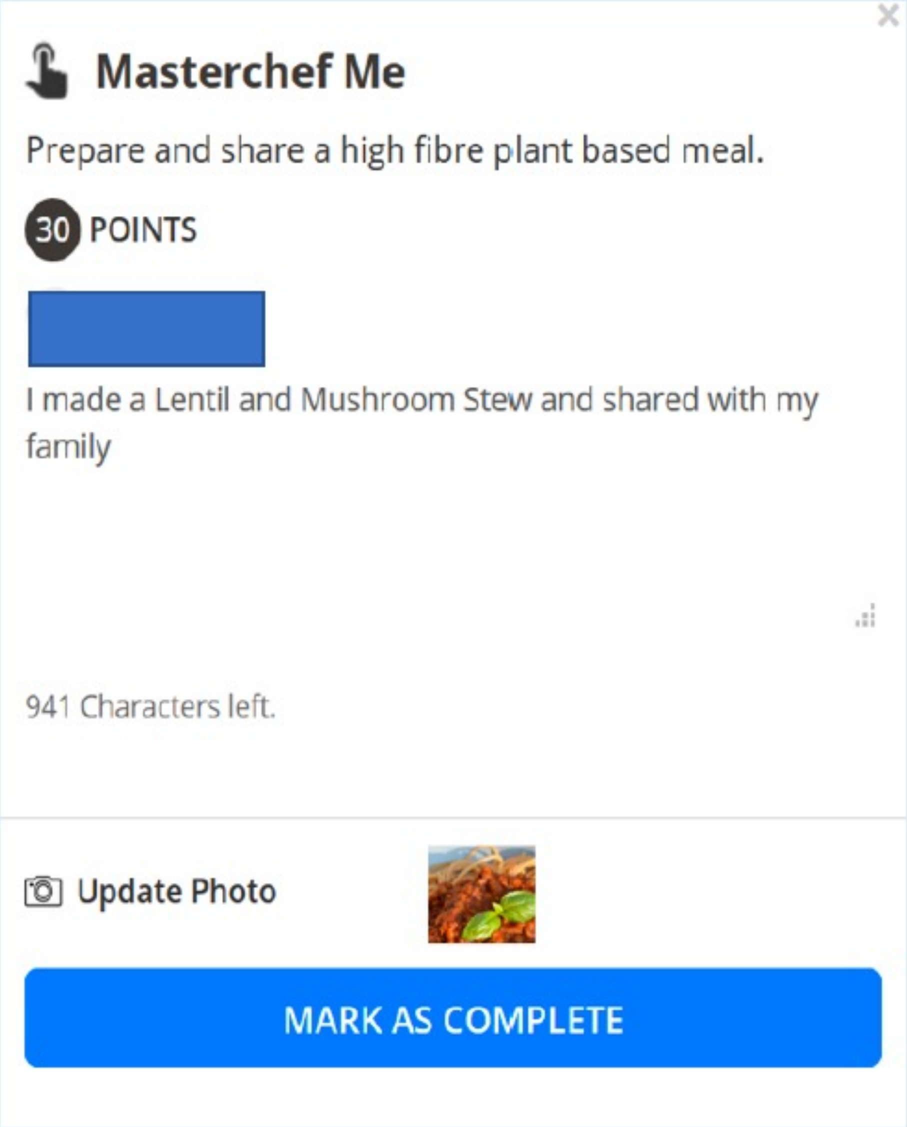

View of Lesson 6 Challenge Log Pages: When participants clicked on the challenge icon (previous image) it opened up a screen to log daily challenges (possibility of 10 points scored daily) and weekly challenges (30 points scored per week). Name and photo ID has been obscured for privacy reasons

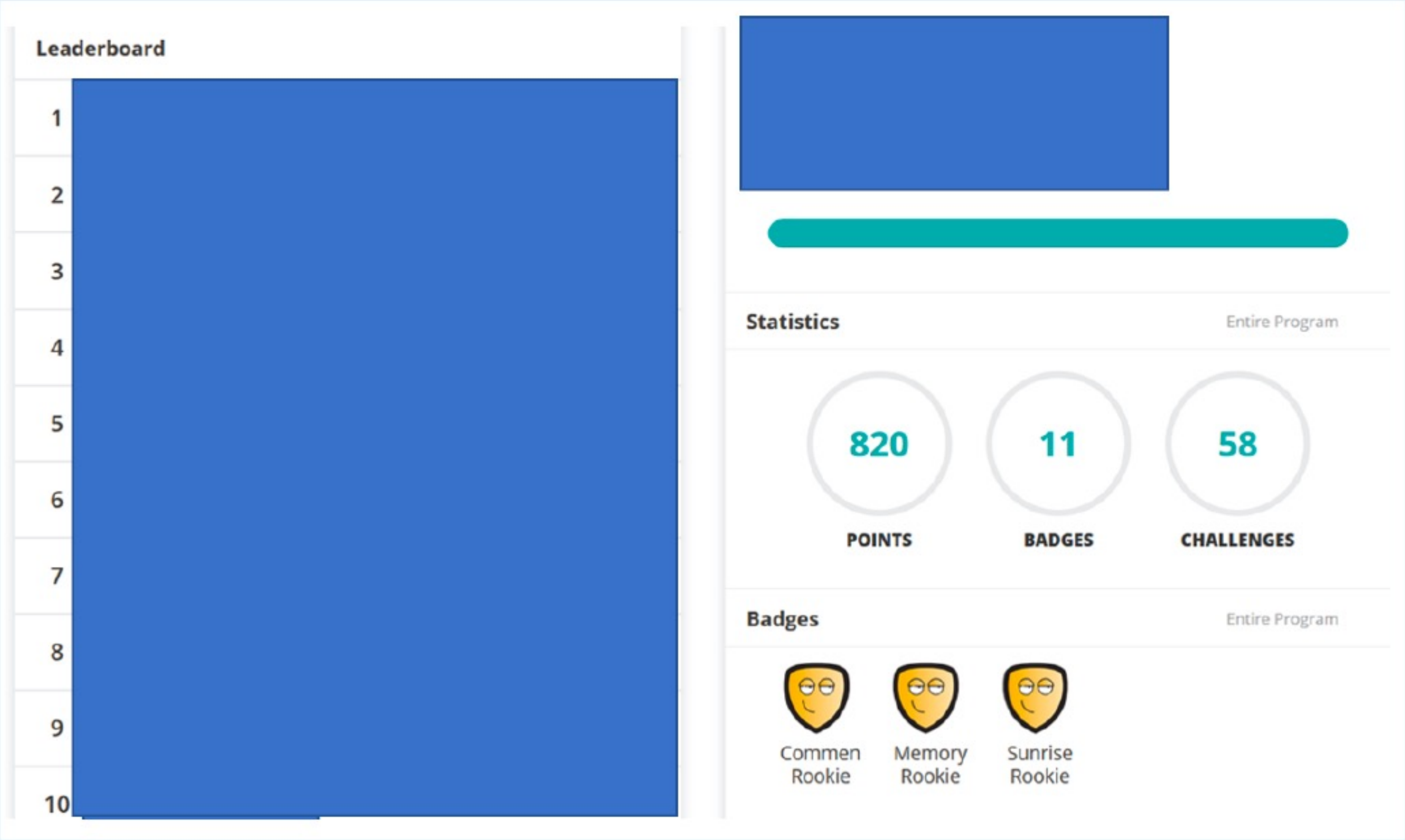

View of Leaderboard Names and photos have been blocked to protect privacy. Participants could compare their scores with others and earn badges for completing activities

Section 2:

The App

The following images are from the mobile app called “mywellness” (2018)

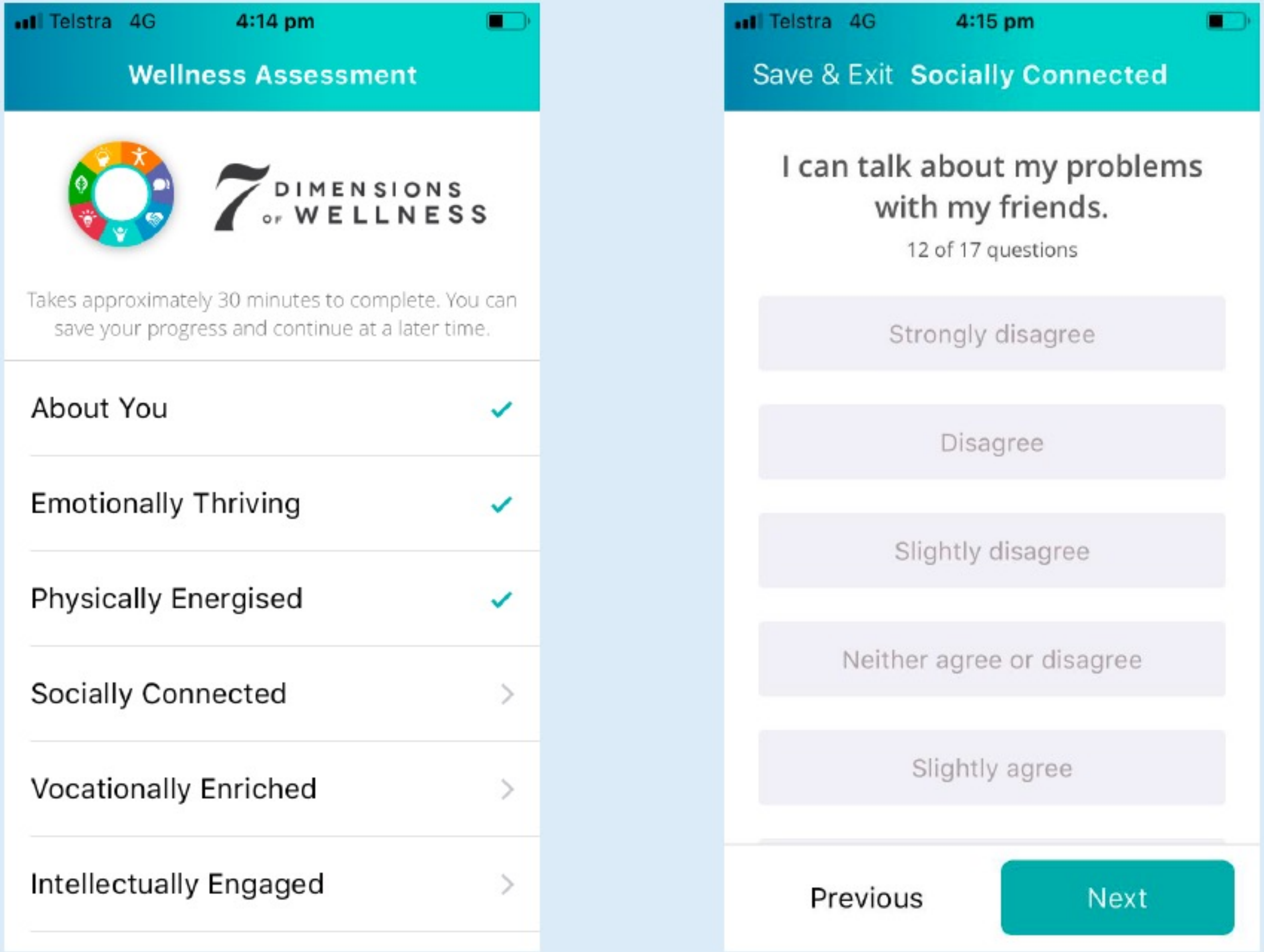

Postquestionnaire sample: No slides are available of the qualitative questions, however participants read the statement about what made it easy or hard to adhere, and were then provided with an expanding text-box to type an answer (length unlimited).

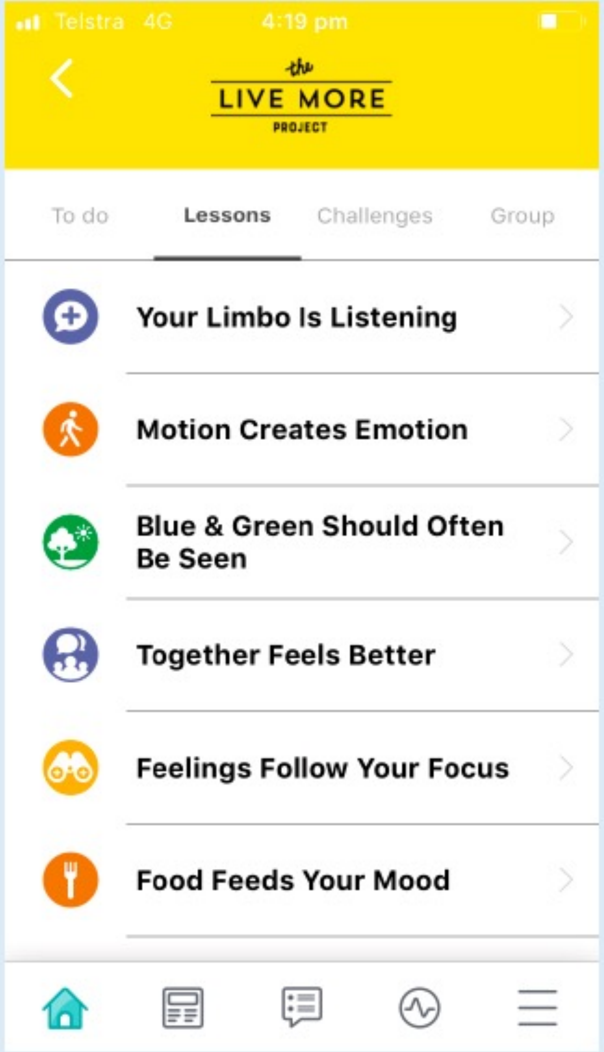

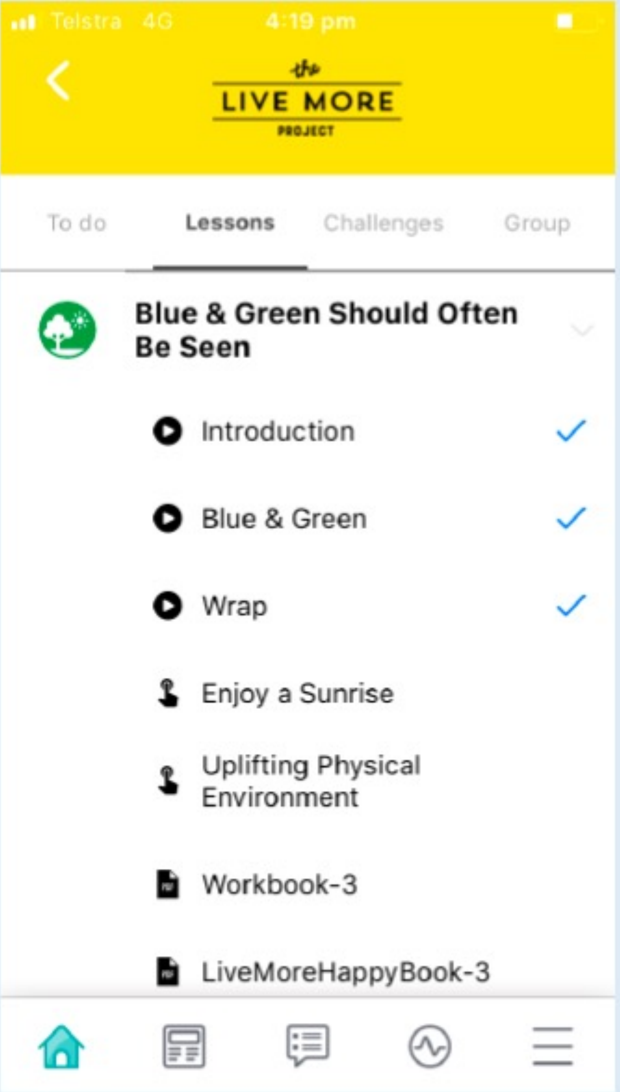

Video content on the mobile app: Participants watched the video content and could download a workbook and a chapter of the accompanying text-book book "Live More Happy" (Morton, 2018) from the Lessons page on the app (or the website).

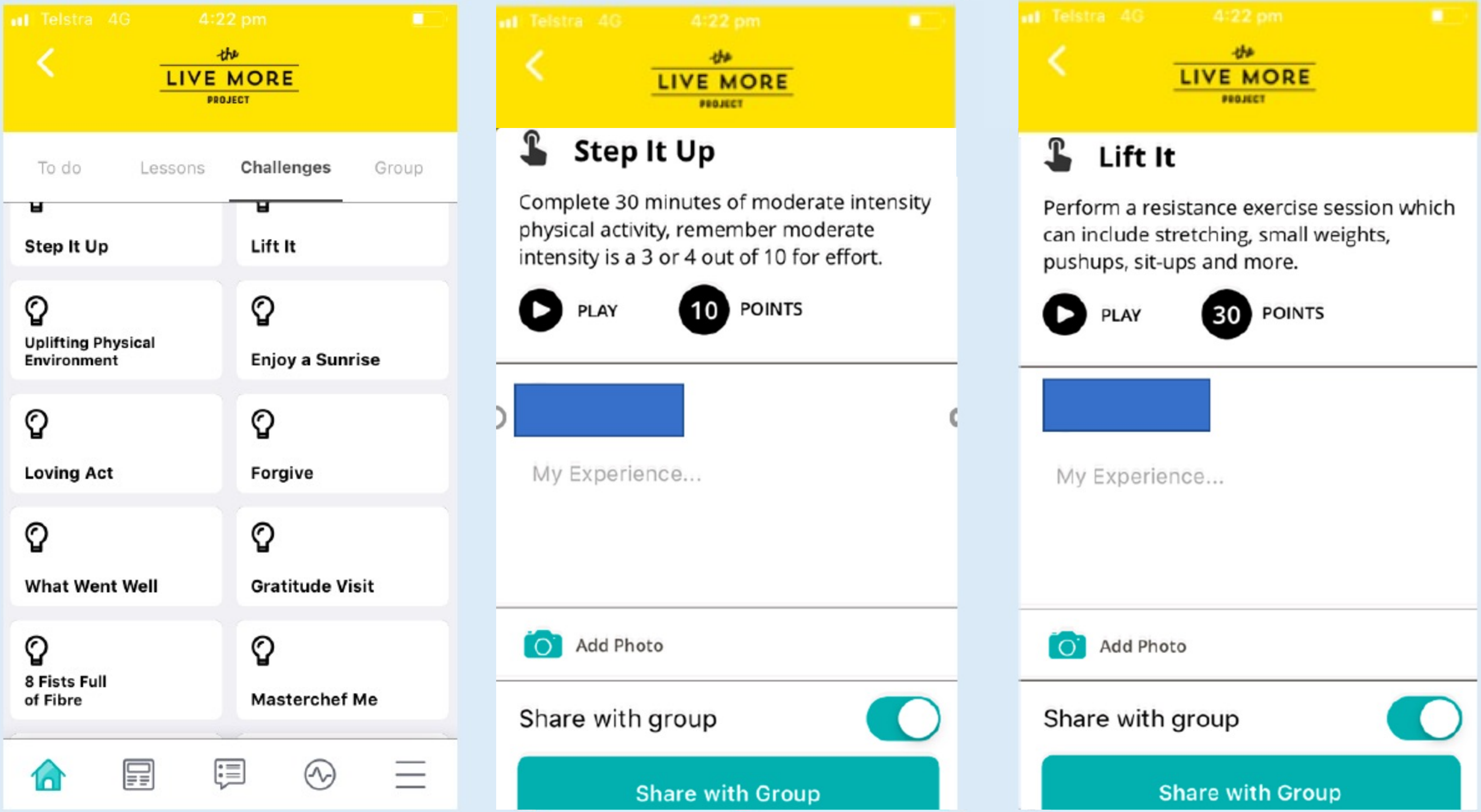

Challenges on the App: Participants could click on the icon and then record challenge activity while 'on the go'. The 'blue' box is simply to de-identify personal information.

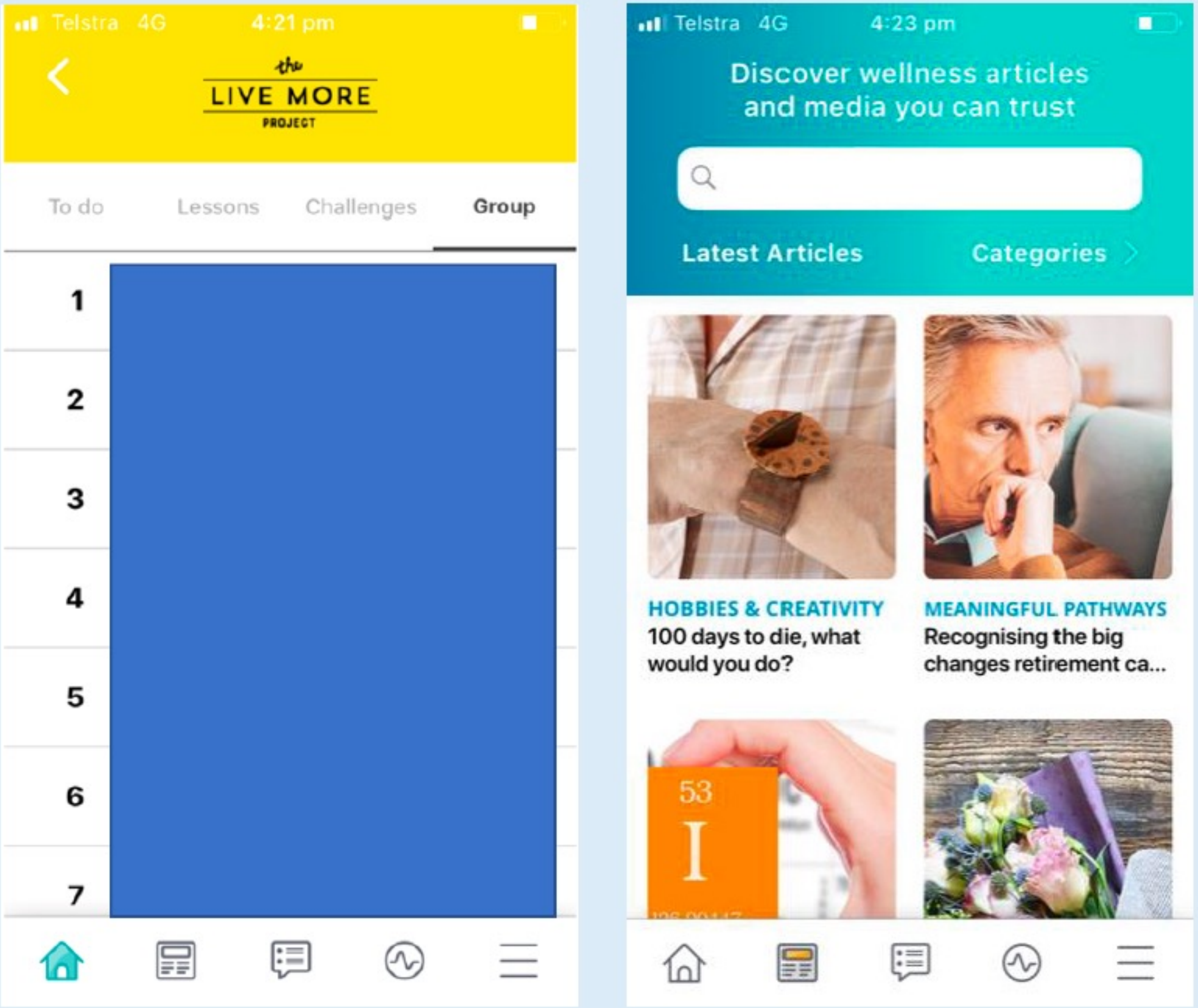

Leaderboard and extra articles: Names and photos have been removed for privacy. Participants could compare their progress with others on the leaderboard and read articles of interest related to the topics of the program.

References

Morton D, Live more happy : scientifically proven ways to lift your mood and your life. Warburton, Vic.: Signs Publishing Company; 2018; ISBN:9781925044720.
